# Design of Plasmonic Yagi–Uda Nanoantennas for Chip-Scale Optical Wireless Communications

**DOI:** 10.3390/s22197336

**Published:** 2022-09-27

**Authors:** Gabriel H. B. Damasceno, William O. F. Carvalho, Jorge Ricardo Mejía-Salazar

**Affiliations:** National Institute of Telecommunications (Inatel), Santa Rita do Sapucaí 37540-000, MG, Brazil

**Keywords:** plasmonic nanoantennas, nanoantennas, nanolinks, Yagi–Uda, optical communications

## Abstract

Optical wireless transmission has recently become a major cutting-edge alternative for on-chip/inter-chip communications with higher transmission speeds and improved power efficiency. Plasmonic nanoantennas, the building blocks of this new nanoscale communication paradigm, require precise design to have directional radiation and improved communication ranges. Particular interest has been paid to plasmonic Yagi–Uda, i.e., the optical analog of the conventional Radio Frequency (RF) Yagi–Uda design, which may allow directional radiation of plasmonic fields. However, in contrast to the RF model, an overall design strategy for the directional and optimized front-to-back ratio of the radiated far-field patterns is lacking. In this work, a guide for the optimized design of Yagi–Uda plasmonic nanoantennas is shown. In particular, five different design conditions are used to study the effects of sizes and spacing between the constituent parts (made of Au). Importantly, it is numerically demonstrated (using the scattered fields) that closely spaced nanoantenna elements are not appropriated for directional light-to-plasmon conversion/radiation. In contrast, if the elements of the nanoantenna are widely spaced, the structure behaves like a one-dimensional array of nanodipoles, producing a funnel-like radiation pattern (not suitable for on-chip wireless optical transmission). Therefore, based on the results here, it can be concluded that the constituent metallic rib lengths must be optimized to exhibit the resonance at the working wavelength, whilst their separations should follow the relation $\lambda_{\mathrm{eff}}/\pi$, where $\lambda_{\mathrm{eff}}$ indicates the effective wavelength scaling for plasmonic nanostructures.

## 1. Introduction

Plasmonic nanoantennas, i.e., high-frequency analogs of Radio Frequency (RF) antennas, can be tailored to operate in the terahertz [1,2,3], infrared [4], and visible frequencies [5] for a plethora of applications, including directive radiation [6], gas sensing [7], biosensing [8], chemosensing [9], photovoltaics [10], electromagnetically induced transparency [11], and optical microscopy [12], among others. In particular, the high-frequency operation of plasmonic nanoantennas promises seamless integration of the future sixth generation of mobile communications networks (6G) into existing fiber-optic infrastructures, crucially important to avoid communication bottlenecks. Such integration uses plasmonic nanoantennas in two different, yet complementary ways: (i) the unique ability of plasmonic nanoantennas to localize electromagnetic fields into deep subwavelength regions is used for direct integration of high-frequency wireless signals into Photonic-Integrated Circuits (PICs) [13,14]; (ii) the radiative properties of plasmonic nanoantennas is exploited for high-speed and broadband nanoscale wireless communication networks, surpassing the intrinsic Ohmic losses of plasmonic waveguides (reducing heating and, consequently, improving power consumption) through reduced light–matter interactions [15,16]. Significantly, this last feature enables PICs with unprecedented miniaturization levels [17]. Furthermore, these nanoantennas can be excited by different mechanisms, namely direct light incidence on the structure [18], electrical excitation/tunability [19,20], guided mode excitation [21,22,23], and the excitation by self-assembled quantum dots [24].

In analogy to RF antennas, dimer [25], dipole [26], monopole [27], and bowtie [28] plasmonic nanoantennas have been widely studied and developed. However, these nanoantenna designs are not appropriate for chip-scale wireless broadcasting. In contrast to long-wavelength RF waves, which can propagate long distances with only minor disturbances, radiated high-frequency (with very short-wavelength) plasmonic fields are strongly affected by the surrounding environment and decay rapidly. A successful approach to overcome this limitation in nanoscale wireless communications consists of designing highly directive nanoantennas such as, for instance, rhombic [29], horn [4], plantenna [21], and Yagi–Uda [30] models, which have demonstrated unidirectional radiation through simple and easily implementable nano-architectures [31,32,33]. In fact, directive nanoantennas have been recently used for lab-on-chip wireless interconnections, including high-speed communication and quantum computing [34,35]. In addition, more complex architectures were proposed on massively heterogeneous processors using optical wireless communications, whose unique terahertz graphene nanoantenna beam reconfigurability demonstrated feasibility for computer architecture communications [2,3]. In the optical domain, on the contrary, reconfigurability at the on-chip wireless interconnection level can be reached through multiple transmitters and receivers using optical-phased antenna arrays [36].

Among all these previous approaches, the Yagi–Uda model stands out for its extraordinary gain and high front-to-back ratio (unidirectional radiation) of the radiated beam. Nevertheless, a general (or optimized) design strategy for Yagi–Uda-like nanoantennas is lacking, i.e., each research group uses its own different design rule for this nanoantenna model [17,37,38]. Over the last years, some approaches to design Yagi–Uda nanoantennas have been proposed. Core-shell (silica-silver) spheres achieved a directivity of only D=3, using eight director elements spaced by λ/1.53 in the visible range [39]. Another Yagi–Uda proposal with eight silver spherical elements was shown in Ref. [40]; the directors were considered spaced by λ/2.7, obtaining D=12.5. An array of single ribs of aluminum with only three director-shown *D* values of up to D=20 when separated by a distance of λ/4 [41]. In the infrared regime, gold nanoribs in a Yagi–Uda arrangement (working at λ=1060 nm) consisting of three director elements separated by λ/4.24 was used to obtain D=3.2 [42]. Hybrid silver-core and silicon-clad nanowires have also been used in the visible wavelength range, providing values of D=17.21 using four directors spaced by λ/4.8 [43].

In this work, five different design approaches for directive plasmonic Yagi–Uda-like nanoantennas are presented and compared. The scattering cross-sections ($\sigma_{\mathrm{sca}}$), far-field directivity (*D*), and footprint parameters were used for analysis. Detailed explanations of the theoretical background are also given, describing the corresponding numerical approach with the COMSOL Multiphysics software. For simplicity, all nanoantenna building components were considered to be made of Au, whilst the nanoantenna is embedded in silica (SiO${}_{2}$). Numerical results were evaluated for the working wavelength λ=1550 nm at the optical C-band to demonstrate the integrability of the concept in this work with conventional integrated optical communication networks. Numerical results indicate that the separation between the successive building elements in plasmonic Yagi–Uda-like nanoantennas should be precisely controlled for efficient light-to-plasmon conversion and re-radiation with high directivity. The main contributions from this work are listed below:

- Comparison among five different plasmonic Yagi–Uda nanoantenna designs for highly directive chip-scale optical nanolinks;
- Plasmonic Yagi–Uda nanoantennas cannot be directly designed following the design rule for their RF antenna counterpart.
- A design strategy for plasmonic Yagi–Uda nanoantennas exhibiting optimized directivity and high integrability with current PICs for optical communications applications;
- The physics behind plasmonic Yagi–Uda nanostructures with: (i) very close; (ii) intermediate; and (iii) very distant elements are elucidated.

The rest of the paper is organized in three different sections as follows. Section 2 describes the characteristics of plasmonic nanoantennas as well as the details of each of the cases studied. Comparative results for the five cases in this work are shown in Section 3, where one can define the better case for chip-scale optical wireless communications. Finally, conclusions from this work are presented in Section 4.

## 2. Theoretical Background

**Figure 1:**
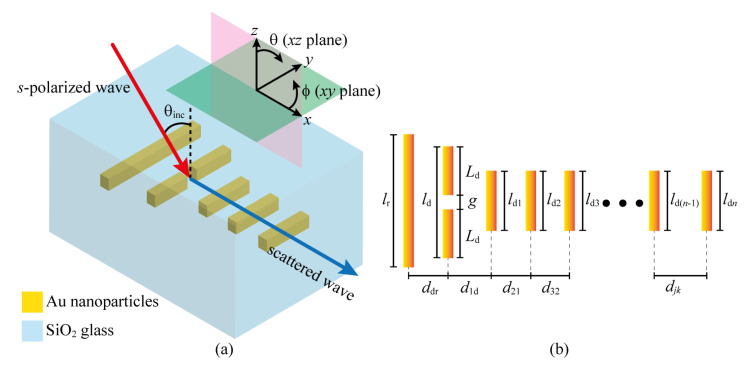
(**a**) Pictorial representation of a generic plasmonic Yagi–Uda nanoantenna (made of Au) embedded in silica. The nanoantenna elements and their geometries are illustrated in (**b**).

Figure 1a depicts a prototypical plasmonic Yagi–Uda nanoantenna, comprising Au nanoribs embedded in SiO${}_{2}$, which can be fabricated using commercially available experimental techniques [44,45,46,47]. All nanoantenna elements have a squared cross-section with a side length of 30 nm. The device is excited by an *s*-polarized wave (illustrated by a red arrow), which can be a laser source or an optical fiber’s end, focused on impinging on the nanoantenna with an angle of $\theta_{\mathrm{inc}}$. Since the main interest is on the signal transmitted by the nanoantenna, the attention will be focused on the analysis of the scattered field, which points out along the *x*-axis, represented by the blue arrow. The scattering efficiency and directivity of the structure depend not only on the relationship between the incident wavelength and the rib lengths (for plasmonic resonances), but also on the corresponding geometrical parameters of the arrangement, i.e., the separation between the nanoantenna elements and the director lengths (which can be the same or different for each of the directors, depending on the design strategy). Therefore, plasmonic Yagi–Uda nanoantennas are first described within a general framework, consisting of three main parts: (i) the reflector, considered with length $l_{r}$; (ii) a dipolar feed (a dipole nanoantenna in this case), built by two plasmonic arms of lengths $L_{d}$ (see Figure 1b), separated by a gap *g*, totaling a length of $l_{d}=2L_{d}+g$; (iii) a set of *n* directors, with lengths $l_{di}$ (with i=1,2,3,…,n), which contribute to improving the directionality of the system. The distance between successive directors is denoted by $d_{jk}$, where j=2,3,4,…,n and k=j−1, as illustrated in Figure 1b. The dipole feed is separated from the reflector by a distance $d_{\mathrm{dr}}$, whilst the first director is placed at a distance of $d_{1d}$ from the dipole feed, also shown in Figure 1b.

Under electromagnetic wave incidence, the free electron charges in the metal dipole feed undergo harmonic oscillations, called Localized Surface Plasmon Resonances (LSPRs), due to the resonant coupling with the incident wave [16]. This resonant feature is achieved by properly designing the nanoantenna elements so that only the dipole feed resonates at the incident wavelength. The reflector consists of a plasmonic element with a resonance far (larger wavelengths) from the dipole feed, which conventionally consists of a rib with the longest length in the structure (as illustrated in Figure 1b). The directors, on the contrary, have sizes similar (or lower) to that of the feed in order to enable a successive near-field coupling which, in consequence, induces near-field directivity in the nanostructure. Therefore, the radiated field (the far-field, in particular) is intrinsically directive due to the field directivity in the structure.

In the space surrounding the plasmonic nanoantenna, there are, in general, total electric ($E_{\mathrm{tot}}$) and magnetic ($H_{\mathrm{tot}}$) fields, which are composed of the superposition of the incident and scattered fields [48], represented by

(1) $$E_{\mathrm{tot}}=E_{\mathrm{inc}}+E_{\mathrm{sca}}$$

and

(2) $$H_{\mathrm{tot}}=H_{\mathrm{inc}}+H_{\mathrm{sca}}.$$

Since the incident field is only of interest for the excitation of the nanoantenna, the attention will be focused on the scattered fields, i.e., the fields produced after the interaction of the incident wave with the nanostructure. An important parameter is the scattering cross-section, calculated as

(3) $$\sigma_{\mathrm{sca}}=\frac{1}{|S_{\mathrm{inc}}|}\oint_{S}S_{\mathrm{sca}}\;ds,$$

which exhibits maximum efficiencies under plasmonic resonant conditions, i.e., maximum $\sigma_{\mathrm{sca}}$ values indicate maximum conversion of the freely propagating waves into LSPRs, as noticed from Equation (3). This latter equation is integrated within a closed surface enclosing the plasmonic nanoantenna, where $S_{\mathrm{inc}}$ and $S_{\mathrm{sca}}$ are calculated using Equations (1) and (2), see Ref. [48] for details.

In contrast to the RF domain, where metals behave as perfect electric conductors, the nanoantenna features are very sensitive to the corresponding plasmonic resonances when working in the optical regime [16]. To simplify the design, the effective wavelength is used ($\lambda_{\mathrm{eff}}$) to scale for plasmonic nanoantennas [49]

(4) $$\lambda_{\mathrm{eff}}=a+b\frac{\lambda_{\mathrm{inc}}}{\lambda_{p}}$$

with *a* and *b* described by

(5) $$\begin{array}{ccc} a & = & -R\left(24+0.75\frac{\varepsilon_{\infty }}{\varepsilon_{s}}\right), \end{array}$$

(6) $$\begin{array}{ccc} b & = & 0.75\frac{R}{\varepsilon_{s}}\sqrt{\varepsilon_{\infty }+141.04\varepsilon_{s}}, \end{array}$$

where R≈15 nm (the approximate radius of the Yagi–Uda elements), $\lambda_{\mathrm{inc}}=1550$ nm (the vacuum incident wavelength), $\lambda_{p}=137$ nm (the bulk plasmon energy, for Au in this case), the high-frequency permittivity $\varepsilon_{\infty }=1$ [50], and the permittivity of the surrounding dielectric environment $\varepsilon_{s}=2.16$ (considering SiO${}_{2}$) [51]. The vacuum incident wavelength $\lambda_{\mathrm{inc}}=1550$ nm becomes $\lambda_{{\mathrm{SiO}}_{2}}=\lambda_{\mathrm{inc}}/\sqrt{\varepsilon_{s}}=1055$ nm in the silica glass, whereas the corresponding effective wavelength is $\lambda_{\mathrm{eff}}=665$ nm.

### Proposed Designs

Using the previous concepts, the five comparison cases in this work will now be defined. Each nanostructure consists of seven Yagi–Uda elements, namely, 1 nanodipole, 1 reflector, and 5 directors (see Figure 1). The first case (labeled Case 1) uses the conventional RF Yagi–Uda design rule [52] for the arrangement of the nanoantenna elements. The second case (labeled Case 2) uses the same dipole length and geometric spacing as Case 1, but adapts the lengths of the directors and the reflector to exhibit maximum $\sigma_{\mathrm{sca}}$ around the incident working wavelength ($\lambda_{\mathrm{inc}}=1550$ nm). In the third case (labeled Case 3), the same rib lengths from Case 2 are used but they are separated by a distance of $\lambda_{\mathrm{eff}}/\pi$. The fourth (labeled Case 4) and fifth (labeled Case 5) cases follow the same rationale as Case 3 but use $\lambda_{{\mathrm{SiO}}_{2}}/\pi$ and $\lambda_{{\mathrm{SiO}}_{2}}/2$, respectively.

The numerical results are obtained from three-dimensional Finite Elements Method (FEM) simulations with the commercial software COMSOL Multiphysics^®^. To avoid spurious numerical reflections at the edges of the structure, a cubic, Perfectly Matched Layer (PML) contour with scattering boundary conditions around the entire model is considered. An optimized mesh size was also used for the accuracy in the numerical results. More specifically, a smaller mesh size of $\lambda_{{\mathrm{SiO}}_{2}}/30$ is considered around the Au nanoparticles, whereas a mesh size of $\lambda_{{\mathrm{SiO}}_{2}}/10$ is used for the surrounding dielectric media.

## 3. Results and Discussion

**Figure 2:**
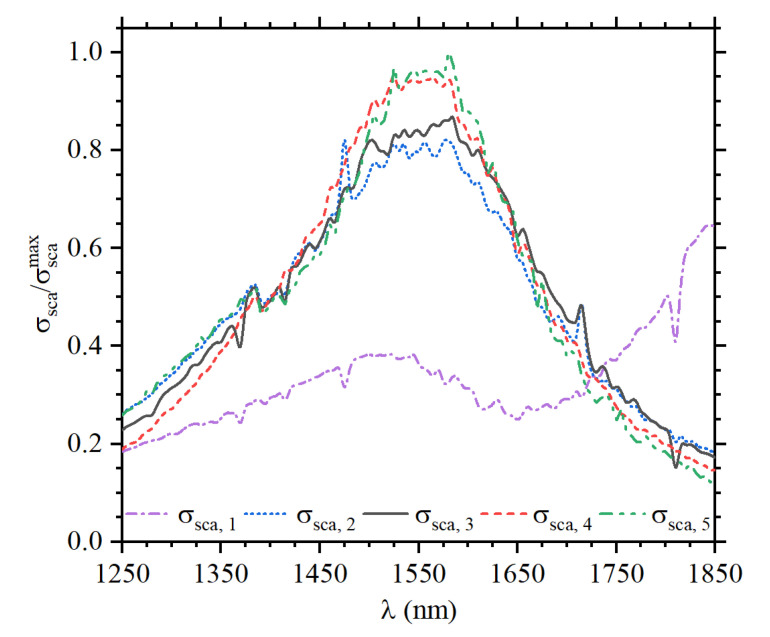
Comparative plot of the scattering cross-section $\sigma_{\mathrm{sca},i}$ (associated with Case *i*) for the optimized designs. The results are normalized in relation to the maximum $\sigma_{\mathrm{sca}}$ value, which was Case 5 in this work.

**Figure 3:**
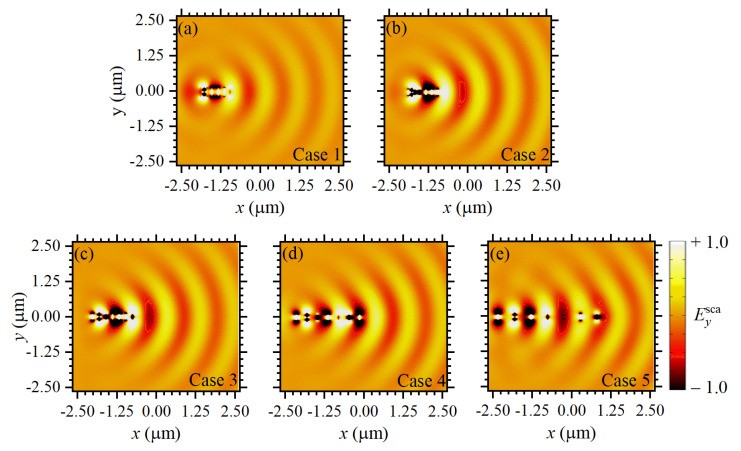
(**a**–**e**) The electromagnetic scattered fields (near-fields) for Cases 1 to 5, respectively.

For nanoantennas with optimized light-to-plasmon coupling, maximizing $\sigma_{\mathrm{sca}}$ (as discussed in the previous section) for the incident working wavelength should be the starting point. In Case 1, following the conventional RF Yagi–Uda design rule [52], the calculations indicate that a nanodipole length $l_{d}=312$ nm exhibits a maximum $\sigma_{\mathrm{sca}}$ at $\lambda_{\mathrm{inc}}=1550$ nm. Interestingly, employing the RF rule for the dipole length calculation $l_{d}=\lambda_{\mathrm{RFmodel}}/2.13$ it was obtained $\lambda_{\mathrm{RFmodel}}=654$ nm, which almost coincides (with a negligible difference of 0.15%) with the effective wavelength $\lambda_{\mathrm{eff}}=665$ nm (calculated in the previous section) for this nanostructure. At first glance, this result indicates that plasmonic Yagi–Uda nanoantennas can be easily designed via the RF rule through effective wavelength scaling. Nevertheless, as observed in Figure 2, this assumption is wrong. In particular, the maximum at $\lambda_{\mathrm{inc}}=1550$ nm for Case 1 is a local rather than a global maximum. Indeed, the global maximum for Case 1 is above λ=1800 nm (not interesting in this work), indicating that plasmonic features of the structure are dominated by a nanorod-like resonance for the system as a whole (see Figure 3a). Moreover, comparing the normalized scattering profiles $\sigma_{\mathrm{sca},i}$ (where i=1,…,5 indicates $\sigma_{\mathrm{sca}}$ for the corresponding Case *i*) for all cases (see Figure 2), it is directly noticed that Case 1 is the most inefficient for converting light-to-plasmon fields at $\lambda_{\mathrm{inc}}=1550$ nm. It is worth remarking that the design approaches for Cases 2 to 5 were described in the previous section. It should also be mentioned that the non-smooth resonance peaks in Figure 2 are due to the sharp rectangular edges of the ribs in the system. In Table 1, for reproducibility and comparison purposes, the optimized geometries for all five cases are thoroughly described.

A deeper understanding of the working principle of the designed nanoantennas can be achieved by observing the corresponding resonant near-fields of the nanostructures. In fact, when working with different but closely spaced plasmonic nanoparticles, plasmon hybridization occurs. The latter term refers to the concept of plasmonic molecules, which, in analogy to the overlap of well-localized atomic orbitals in molecules, depends on the overlap of plasmonic near-fields between adjacent metallic nanoparticles. Figure 3a–e shows the scattered electric field ($E_{y}^{\mathrm{sca}}=E_{y}^{\mathrm{tot}}-E_{y}^{\mathrm{inc}}$) along the plane of each nanoantenna, i.e., the xy-plane for all of the five cases (normalized in relation to Case 5). These results were calculated using an incident angle of $\theta_{\mathrm{inc}}=60^{\circ }$. The system in Figure 3a has a total length of around 1000 nm, which is the order of $\lambda_{{\mathrm{SiO}}_{2}}$ (calculated above), making the system behave as an effective nanorod (as a whole). Importantly, the near-field symmetry observed in Figure 3a and the corresponding $\sigma_{\mathrm{sca},1}$ (in Figure 2) are in excellent qualitative agreement with plasmonic resonances for a nanorod [53], reinforcing the plasmon hybridization assumption. For Cases 2 to 5, in Figure 3b–e, where the distance between the building elements is successively increased, it was observed that the symmetry of the electromagnetic near-field distributions change. The field profile in Figure 3e, for Case 5, indicates an array of dipoles, i.e., each building element has a nearly isolated dipolar resonance. In contrast, Figure 3b–d for Cases 2 to 4, respectively, demonstrate collective oscillations through the large near-field overlap between the successive nanoantenna elements.

**Figure 4:**
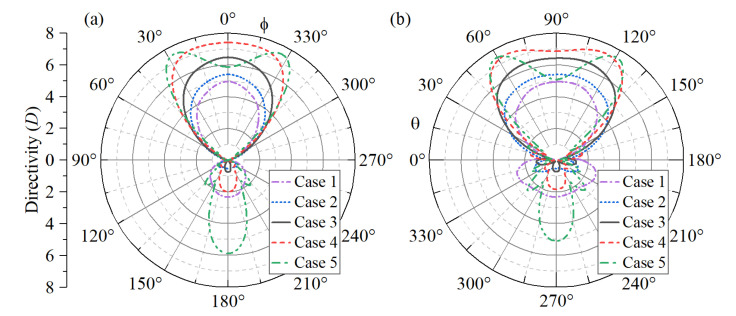
Far-field radiation patterns for the five scenarios in (**a**) azimuth and (**b**) elevation.

Since the focus of this work is the design of nanoantennas for wireless optical broadcasting at the chip-scale level, the corresponding far-field radiation properties for the five cases will be analyzed. Figure 4a,b comparatively shows the corresponding far-field radiation patterns for the azimuth and elevation angles, respectively. The nanoantennas in this figure were excited by an incident plane wave directly impinging on the nanostructure, with an incident angle $\theta_{\mathrm{inc}}=60^{\circ }$. The directivity is calculated using the conventional expression [52]:

(7) $$D\left(\phi ,\theta \right)=\frac{F(\phi ,\theta )}{\frac{1}{4\pi }\int_{0}^{\pi }\int_{0}^{2\pi }F\left(\phi ,\theta \right)\sin\left(\theta \right)\;d\theta \;d\phi },$$

where F(ϕ,θ) is a function of the radiation intensity. As expected from previous discussions, Case 1 exhibited the worst far-field performance. Case 2 also exhibits poor far-field radiation in comparison with Cases 3 to 5. Although Case 5 has the most efficient far-field radiation, the main radiation lobes are tilted in relation to the plane of the nanoantenna. The same occurs for Case 4 in the elevation plane. Therefore, it can be concluded that the design based on Case 3 has the best broadcasting performance, considering the transmission along the axis of the nanoantenna (*x*-axis), as observed in Figure 4a,b. It should also be noted that Case 3 shows the best front-to-back ratio in the far-field patterns.

**Figure 5:**
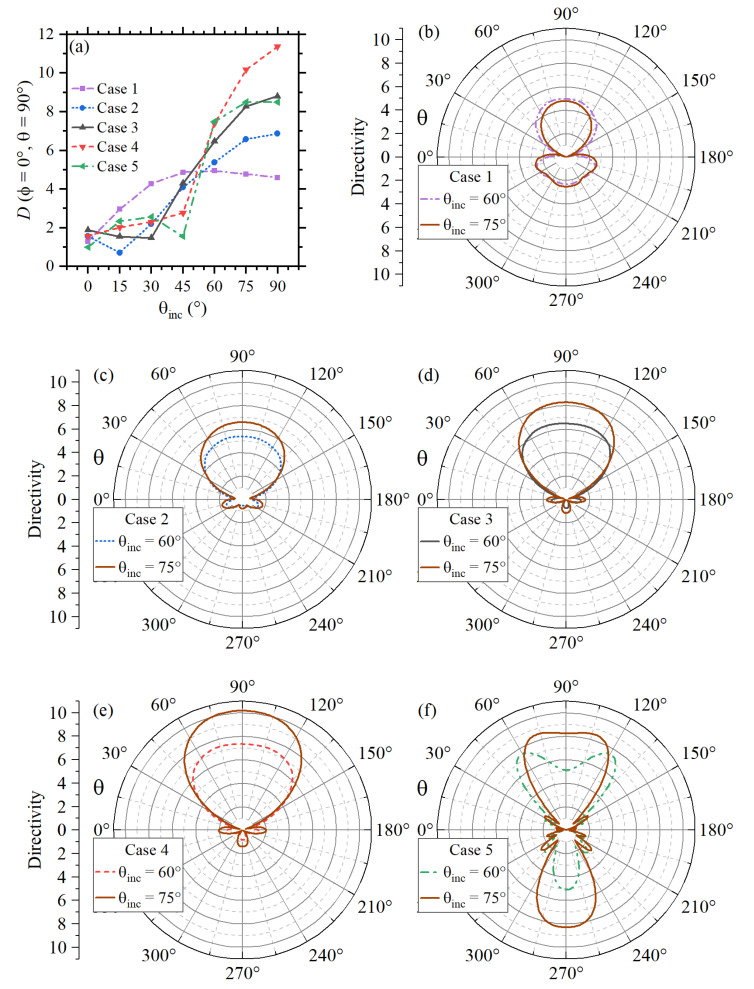
(**a**) Comparative analysis of directivity towards +x-axis [$D(\phi =0^{\circ },\theta =90^{\circ })$], as a function of $\theta_{\mathrm{inc}}$, for all five cases. Numerical results of the corresponding far-field patterns for $\theta_{\mathrm{inc}}=60^{\circ }$ and $\theta_{\mathrm{inc}}=75^{\circ }$ are comparatively plotted in (**b**–**f**) for all five cases, respectively.

**Figure 6:**
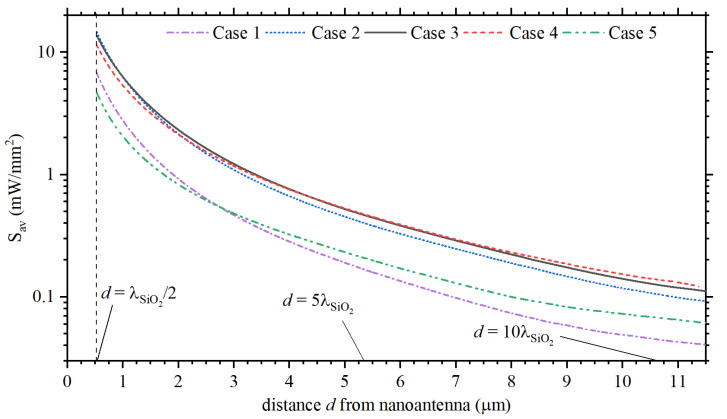
Numerical results for Sav=(1/2)Re(Esca×Hsca*) along the *x*-axis. Data were collected for $d\geq \lambda_{{\mathrm{SiO}}_{2}}/2$, considering d=0 as the last director for each design.

The calculations in Figure 4 were made for $\theta_{\mathrm{inc}}=60^{\circ }$. Nevertheless, the incident angle also affects the directivity along the *x*-axis. In fact, the best results are expected for $\theta_{\mathrm{inc}}=90^{\circ }$, as noticed from the numerical results for Cases 3 and 4 in Figure 5a, where the maximum directivity is achieved for each case. However, conventional PICs are fed by the end of the tilt optical fiber. Therefore, to be consistent with the current developments in PICs, the far-field radiation patterns for Cases 1 to 5 are compared in Figure 5b–f, respectively, considering $\theta_{\mathrm{inc}}=60^{\circ }$ and $\theta_{\mathrm{inc}}=75^{\circ }$. It should be remarked that results for $\theta_{\mathrm{inc}}=75^{\circ }$ and $\theta_{\mathrm{inc}}=90^{\circ }$ only have a negligible difference, see Figure 5a. To emphasize the feasibility of the optimized design in this work, the time-averaged Poynting vector, namely, Sav=(1/2)Re(Esca×Hsca*) was calculated along the *x*-axis, as shown in Figure 6, for all five cases. The feed laser light is considered with an incident angle $\theta_{\mathrm{inc}}=60^{\circ }$. Since we are interested in the energy radiated into the far-field region, calculations in this last figure were made at a distance of $d\geq \lambda_{{\mathrm{SiO}}_{2}}/2$ from the last director (defined as d=0) of each design. The numerical data for the propagation losses are in qualitative agreement with what is expected from the Friss equation. Moreover, the results for $S_{\mathrm{av}}$ indicate the possibility of developing optical wireless nanolinks separated by distances *d* that are several times larger than $\lambda_{{\mathrm{SiO}}_{2}}$, which are in agreement with the far-fields in Figure 4 (specifically for $D(\phi =0^{\circ },\theta =90^{\circ })$).

**Figure 7:**
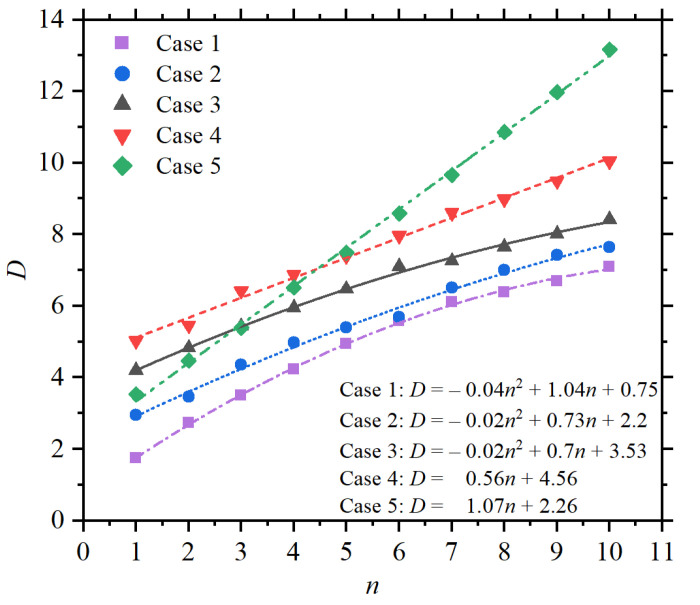
Directivity *D* as a function of the number of directors *n* for Cases 1 to 5.

**Figure 8:**
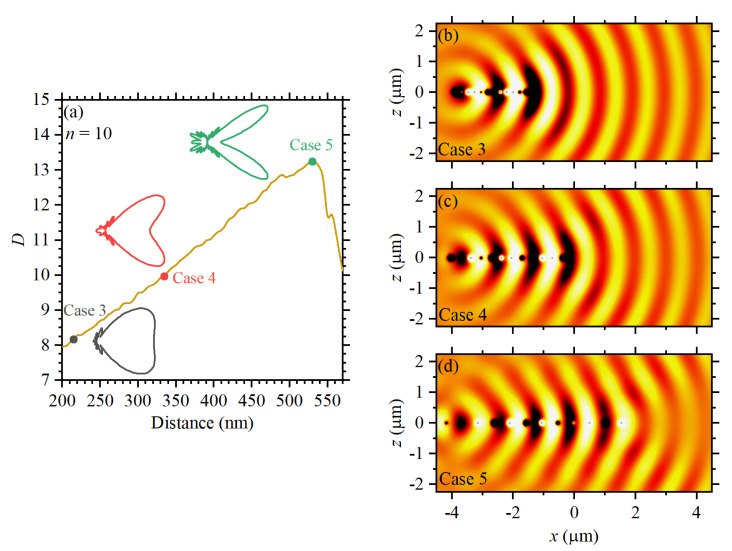
(**a**) Directivity as a function of the inter-particle distances for Cases 3 to 5 corresponding to n=10. (**b**–**d**) Show the corresponding scattered fields (around the structure) for each case.

For completeness of the analysis, the directivity (*D*) as a function of the number (*n*) of directors for all five cases was also studied. Numerical data for *D* as a function of *n* is shown in Figure 7 for Cases 1 to 5, considering *n* from 1 to 10. From these results, it can be seen that *D* increases linearly with *n* for Cases 4 and 5, whereas a parabolic increase is observed for Cases 1 to 3, as shown by the corresponding fitting equations in the inset. Though the best *D* values from Figure 7 are noticed for Cases 4 and 5, the funnel-like profile of the far-field for these cases should be considered. Indeed, the funnel-like profile becomes remarkable for relatively large *n*-values, as demonstrated in the insets in Figure 8a, where *D* is plotted as a function of the inter-particle distances for Cases 3 to 5, considering n=10. The normalized scattered field profiles along the elevation plane (i.e., the xz-plane) are shown in Figure 8b–d, which indicates that the best front-to-back ratio, with the best signal transmission towards the front of the antenna, corresponds to Case 3. Compared with other strategies to design plasmonic Yagi–Uda nanoantennas with spherical [38], ribs [37], or different shapes [17], the concept in this work offers a mechanism for improved directivity in optical wireless nanolinks through a simple, feasible, and easily implementable design.

## 4. Conclusions and Outlook

In summary, it was demonstrated that a design strategy for plasmonic Yagi–Uda nanoantennas works at $\lambda_{\mathrm{inc}}=1550$ nm, with optimized far-field radiation patterns and directivity. In doing so, five different design approaches were comparatively studied where, in addition to the far-field analyses, the corresponding near-fields were used to explain the physical mechanism behind each design. Indeed, it was numerically demonstrated that the concept of plasmonic hybridization, i.e., the near-field overlap of closely spaced plasmonic nanoparticles, plays a crucial role in light-to-plasmon conversion and radiation. In particular, it was numerically observed that there are two limiting cases: (i) when the building elements are closely spaced, the nanoantenna tends to behave as an effective nanorod (as a whole); and (ii) when the building elements are widely spaced, the system starts working similar to a one-dimensional arrange of radiating nanodipoles. From the results here, it can be concluded that $\lambda_{\mathrm{eff}}/\pi$ ( where $\lambda_{\mathrm{eff}}$ refers to the effective wavelength scaling for plasmonic nanostructures), corresponding to Case 3 in the analyses, produces the best far-field radiation pattern with an optimized front-to-back ratio. It is worth mentioning that $\lambda_{\mathrm{eff}}$ depends on the incident wavelength, the plasmonic properties of the metallic building material, the dielectric properties of the surrounding medium, and the cross-section of the ribs, indicating that the model can be extended to other working wavelengths and materials. The design conceptualized in this work can be easily implemented by experimentalist researchers to provide new and efficient directive nanoantennas for optical wireless broadcasting in future PICs.

## Figures and Tables

**Table 1:** Optimized geometric parameters for the five cases.

Lengths (nm)	Case 1 (RF Design Rule Using $\lambda_{\mathrm{eff}}$)	Case 2 (Optimized Lengths)	Case 3 (Optimized Lengths)	Case 4 (Optimized Lengths)	Case 5 (Optimized Lengths)
$l_{r}$	348	194	194	194	194
$l_{d}$	312	312	312	312	312
$l_{d1}$	277	194	194	194	194
$l_{d2}$	273	194	194	194	194
$l_{d3}$	275	194	194	194	194
$l_{d4}$	277	194	194	194	194
$l_{d5}$	275	194	194	194	194
**Distances** **(nm)**	**Case 1** **(RF Design Rule** **Using $\lambda_{\mathrm{eff}}$)**	**Case 2** **(RF Design Rule** **Using $\lambda_{\mathrm{eff}}$)**	**Case 3** **(Distances of** $\lambda_{\mathrm{eff}}/\pi$)	**Case 4** **(Distances of** **$\lambda_{{\mathrm{SiO}}_{2}}/\pi$)**	**Case 5** **(Distances of** **$\lambda_{{\mathrm{SiO}}_{2}}/2$)**
*g*	10	10	10	10	10
$d_{\mathrm{dr}}$	114	114	213	337	529
$d_{1d}$	94	94	213	337	529
$d_{21}$	147	147	213	337	529
$d_{32}$	208	208	213	337	529
$d_{43}$	188	188	213	337	529
$d_{54}$	188	188	213	337	529

## Data Availability

Data underlying the results presented in this paper are not publicly available at this time but may be obtained from the authors upon reasonable request.
